# Green management training program and its effect on staff nurses’ organizational citizenship behavior

**DOI:** 10.1186/s12912-025-03203-9

**Published:** 2025-05-19

**Authors:** Nada Salah Yassin, Dalal Talaat Akel, Hanaa Mohamed Abd Rabou

**Affiliations:** 1 Nursing Administration, Faculty of Nursing, Ain Shams University, Cairo, Egypt; 2 Nursing Administration, Faculty of Nursing, Ain Shams University, October 6 University Cairo-Egypt, Cairo, Egypt

**Keywords:** Green management, Organizational citizenship behavior, Training program and staff nurses

## Abstract

**Background:**

Green management practices are essential for organizational sustainability, and their adoption in hospitals can help reduce environmental impact. Organizational citizenship behavior further enhances green management efforts.

**Aim of the study:**

This study aimed to assess the effect of a green management training program on staff nurses’ organizational citizenship behavior.

**Design:**

Quasi-experimental design with a pretest-posttest approach.

**Setting:**

This study was carried out in Al-Ahrar Teaching.

**Subjects:**

85 staff nurses participated in the study.

**Tools of data collection:**

green management knowledge questionnaire, employee green behaviors scale, and organizational citizenship behavior scale were used for data collection.

**Result:**

Significant improvements were observed in staff nurses’ knowledge of green management (16.5–89.4%), employee green behavior (5.9–64.7%), and organizational citizenship behavior (7.1–70.6%) at pre- and post- phases respectively.

**Conclusion:**

The green management training program effectively improved organizational citizenship behavior, supporting the research hypothesis.

**Trial registration number:**

Not applicable.

**Clinical trial number:**

Not applicable.

## Introduction

Green management is the organization-wide process of utilizing innovation to attain sustainability, decreased waste, social responsibility and a competitive advantage. This is achieved through constant enhancement and learning, as well as the adoption of environmental strategies and goals that are fully integrated with the organization’s strategies and goals [1]. Green management in healthcare has emerged as a crucial strategy for promoting environmental sustainability while simultaneously enhancing the operational efficiency and effectiveness of healthcare organizations. According to Healthcare Without Harm, a green hospital actively works to minimize its environmental impact and mitigate health risks associated with environmental degradation [2].

The Green Management Training Program (GMTP) is a central component in implementing green management strategies within healthcare organizations. The GMTP is designed to equip healthcare professionals with the necessary knowledge, skills, and attitudes to foster sustainable practices across their daily operations. Its objectives are to promote environmental sustainability, enhance green behaviors among healthcare staff, and integrate sustainable practices into the organizational culture [2]. By creating a structured training program, hospitals can ensure that all employees, including nurses, understand their role in reducing the organization’s environmental footprint, which ultimately leads to a greener, healthier environment for patients and staff [3].

The structure of the GMTP involves a phased approach to training, beginning with an initial assessment of current organizational practices. This is followed by targeted training sessions that incorporate a mix of in-person workshops, online modules, and practical exercises tailored to specific job roles. These training sessions focus on promoting sustainable behaviors, such as waste reduction, energy efficiency, and sustainable procurement [4]. Furthermore, the GMTP includes training for staff nurses to ensure that they are equipped to foster a culture of environmental responsibility among their colleagues. Ongoing feedback mechanisms and continuous support are also key to the program’s success, ensuring that sustainability practices are maintained and improved over time [5].

Recent studies show that green management practices not only reduce environmental impacts but also improve operational efficiency and organizational performance. For example, sustainability-focused strategies, such as energy efficiency and waste reduction, have been found to correlate with financial savings and improved patient outcomes [6]. Training programs like the GMTP play a significant role in promoting sustainable behaviors among healthcare professionals. They are particularly effective when integrated with leadership initiatives and continuous support systems. In addition to these environmental and operational benefits, GMTP initiatives can also enhance organizational citizenship behavior (OCB), as nurses and staff are motivated to engage in sustainability efforts when they see a clear organizational commitment to these values [7–8].

A critical aspect of green management is the promotion of green behavior, which is defined as actions consistent with the objectives of environmentally sustainable development. As [9] highlight, green behavior is a key strategy for achieving long-term sustainability within healthcare organizations. The green behavior of employees is heavily influenced by the positive green behavior demonstrated by leaders [10], underscoring the importance of leadership in fostering a culture of sustainability. Research suggests that employees who are motivated, included, and well-trained are more likely to engage in environmentally sustainable behaviors [11]. Subsequently, encouraging green behavior among staff nurses, patients, and visitors is very important. By promoting green behavior among staff, patients, and visitors, hospitals can help to diminish their environmental impact & contribute to a healthier, more sustainable future. Green management practices and green behavior in hospitals have positive health outcomes [12].

OCB helps the employees to feel more in control over their jobs and activities and feel noble about helping others. It can be assessed by evaluating how often employees show discretionary and extra-role behaviors [13]. The implementation of the GMTP is expected to enhance OCB by fostering a work environment where sustainability is deeply embedded within the organizational culture. This type of environment encourages employees to feel more empowered and motivated to engage in behaviors beyond their formal responsibilities, which, in turn, enhances the organization’s environmental performance [14]. Furthermore [15] and [16], stress the significance of cultivating an environmental culture within organizations, which drives sustainability objectives through employees’ voluntary actions.

While the connection between green management practices and OCB is often assumed. According to Value-Belief-Norm (VBN) theory, personal values, such as environmental concern, shape beliefs like the New Ecological Paradigm (NEP), which increases awareness of environmental impacts. This awareness, coupled with a sense of individual responsibility, motivates individuals to adopt eco-friendly behaviors [17]. In addition, Social Exchange Theory suggests that employees are more likely to engage in green behaviors when they perceive personal rewards in return. Organizational incentives such as financial rewards, recognition, or other tangible benefits can motivate employees to adopt pro-environmental behaviors, viewing them as part of a social exchange that offers personal benefits [18].

Green management can significantly contribute to OCB among nurses by fostering a positive work environment and enhancing employee engagement. By integrating sustainability practices into daily routines, nurses are encouraged to actively participate in initiatives that align with environmental goals, which fosters a shared sense of purpose [19–20]. This sense of collective responsibility strengthens collaboration and teamwork among colleagues. As nurses become more involved in sustainability efforts, it boosts morale, increases job satisfaction, and leads to higher levels of commitment to organizational goals. Ultimately, this engagement enhances overall organizational performance, increase OCB and improves both patient care and the well-being of healthcare staff [21].

## Aim of the study

The aim of the study is to assess the effect of a green management training program on staff nurses’ organizational citizenship behavior.

### Specific objectives

- To assess staff nurses’ knowledge regarding hospital policies of green management (pre-post-follow-up).
- To assess staff nurses’ green behavior (pre-post-follow-up).
- To design the green management training program.
- To implement the green management training program.
- To assess staff nurses’ organizational citizenship behavior(pre-post-follow-up).
- To Find out the effect of a green management training program on staff nurses’ organizational citizenship behavior.

### Research hypothesis

1. Staff nurses’ knowledge regarding hospital policies will be improved after implementing a green management training program.
2. Staff nurses’ green behavior will be improved after implementing a green management training program.
3. Staff nurses’ organizational citizenship behavior will be improved after implementing a green management training program.

### Methods

A quasi-experimental (one-group pretest/posttest) design was utilized to conduct this study. The study was conducted at Al-Ahrar Teaching Hospital, which is located in Zagazig City, Al-Sharkia Governorate, Egypt. This hospital is affiliated with the general organization for teaching hospitals and institutes. Its total capacity is 420 beds. The study was conducted at noncritical units (surgical, ENT, chest, pediatric, obstetric, oncology, and gastro units). Selecting units using a random technique involves several steps to ensure that the selection is unbiased and representative. Use a random number generator to select numbers corresponding to units on the list using an online tool that generates random numbers within a specified range. Ensure the number of randomly generated numbers matches the desired sample size of units. Match the randomly generated numbers to the units’ unique identifiers.

### Sampling

The study subjects consisted of 85 staff nurses out of 110 staff nurses working in different units at the aforementioned study setting at the time of the study. A simple random sampling technique was applied by drawing lots from the eligible staff nurses. As well as having corresponding to the following inclusion criteria preferred in the current study: Staff nurses with at least more than two years of experience with the current position and willingness to participate in the study were included in the study. Also, nurses who were available at the time of the study. According to this formula, the sample size was calculated [22]:

$$\:\text{n}=\frac{N\times\:p(1-p)}{[N-1\:\times\:(d^2\div\:Z^2)]+\:p(1-p)}\:$$

Where:

n = sample size.

N = total size (110 nurses)

Z = 1.96

d = error level 5%

*p* = 0.5

### Tools of data collection

Three tools have been utilized for collecting data, namely the green management knowledge questionnaire, the employee green behaviors scale, & the organizational citizenship behavior scale.

### Green management knowledge questionnaire

It aimed to evaluate knowledge of staff nurses with regard to green management. It comprised of 2 parts: — first part: It pertained to collecting staff nurses’ personal and job characteristics like sex, age, marital status, qualifications, years of experience, position, & attended training courses. Second part: It aimed to assess staff nurses’ knowledge about green management. It has been developed by the researcher guided by [23–26] and validity and reliability testing were conducted to ensure the strength of the tool. This part was included (35 questions MCQ) which covering different aspects of green management, (Ex, Green management definition, importance of green management, principle of green management, effective activities for green management).

A way to give points or marks to measure achievements. The answers of green management knowledge items were scored (one) for the correct answer and (zero) for the incorrect answer. The total grade was 35. The score of items were summed up and the total was divided by the number of the items. These scores converted into percent score; The total green management knowledge score was categorized as follows: Staff nurses with a knowledge level greater than or equal to 60% were considered to indicate a satisfactory level of knowledge. A score less than 60% was considered an unsatisfactory level of knowledge [24, 25].

### Employee green behaviors scale

This tool has been adapted from [27] and altered by the researcher and validity and reliability testing were conducted to ensure the strength of the tool. It aimed to evaluate staff nurses’ green behaviors. It comprised of twenty-four items (Ex, I suggest my colleagues to engage in environmentally responsible behaviors, I set the computer monitors in sleep mode or turn it off when not in use), which grouped under six categories, with 4 items for each dimension as follows: Environmental Awareness (4 items), Taking Initiative (4 items), Working Sustainably (4 items), Influencing Others (4 items), Avoiding Harm (4 items), and Conserving (4 items).

The answers from staff nurses were rated as follows: - responses have been assessed on a five-point Likert scale, with the options being Always (5), Often (4), Sometimes (3), Rarely (2), and Never (1). These scores have been converted to a percentage score. Furthermore, the mean & standard deviation were computed. The total score is classified as high level if score more than 70% and moderate if total score ranged between from (50–70%) and low if score was less than 50% [27]. The internal consistency reliability of the scales was evaluated using Cronbach’s alpha for Environmental Awareness, taking initiative, Influencing other, Avoiding harm, Conserving and Working sustainability are 0.823, 0.799, 0.861, 0.834, 0.850 and 0.806 respectively.

### Organizational-citizenship behavior scale

It aimed to assess organizational citizenship behavior between staff nurses. This scale was adopted from [28] based on [29]. It consisted of twenty items (Ex, I collaborate with my manager to perform the duties in best way, I help and guides patients to receive the excellent service), which separated into five dimensions, as follows: Altruism deviant (5 items), Civility deviant (4 items), Sportsmanship deviant (3 items), Civic virtue deviant (5 items), and Conscientiousness deviant (4 items).

Responses of staff nurses have been assessed on a five-point Likert scale, with 1 denoting strongly disagree, 2 denoting Disagree, 3 denoting uncertainty, 4 denoting agreement, & 5 denoting strong agreement. These scores have been changed to a percentage score. Furthermore, the mean and standard deviation were computed. If the total percent score exceeded 70%, OCB was deemed to be high, moderate if it fell within the range of 50 to 70%, and low if it was below 50% [28]. The internal consistency reliability of the scales was evaluated using Cronbach’s alpha for Altruism deviant, Civility deviant, Sportsmanship deviant, Civic virtue deviants and Conscientiousness deviant are 0.796, 0.812, 0.822, 0.901and0.808 respectively.

### The tools validity and reliability

The instruments’ validity has been assessed by a jury panel of six experts (two professors and four assistant professors) from the Nursing Administration Department and Psychiatric Nursing Department of the Faculty of Nursing, Ain Shams, Cairo, Egypt for content and face validation. The instruments have been evaluated for their applicability, comprehensiveness, and relevance. They have been asked to provide their opinions on the proposed instruments. Subsequent to their recommendations, certain items were incorporated or excluded.

The reliability of the data collection tools was assessed its internal consistency by using Cronbach’s Alpha Coefficient test. Green management knowledge questionnaire was 0.846, Employee Green Behaviors Scale was 0.910 and Organizational citizenship behavior Scale was 0.907.

### Ethical consideration

The Scientific Research Ethical Committee of the Faculty of Nursing, Ain Shams University, granted ethical permission under (code number: NUR 24.01.207). The Faculty of Nursing submitted official letters to the designated hospital in order to obtain permission for data collection. Following a thorough explanation of the investigation’s objectives and procedures, each participant (staff nurse) provided informed written consent. The right of all participants to refuse or withdraw from the study at any time was ensured. The complete confidentiality and anonymity of any information that has been obtained were ensured. No actual or possible harm has been predicted from the investigation’s maneuvers.

### Pilot study

Prior to gathering data, a pilot study was carried out. The study tools were filled in a range of twenty to twenty-five minutes. In October 2023, a pilot study has been carried out on eight staff nurses, which constitutes 10% of the research’s sample. The eight nurses were incorporated into the main research group without any modifications; the data obtained from the pilot study has been analyzed.

### Fieldwork

The actual fieldwork of the study lasted for a period of ten months, starting from the commencement of September, 2023, to the end of June, 2024. The investigation has been performed through the following five phases: preliminary phase, planning phase, program implementation phase, evaluation phase, and follow-up phase.

### Phase I (preliminary)

The researcher met with the nursing director of the hospital to determine the appropriate time for gathering the data following obtaining the official permissions for the investigation. The researcher conducted a meeting with all staff nurses to provide an explanation of the research’s objective and nature, as well as to obtain their written consent to participate in the investigation. The data that has been collected was regarded as the baseline or pretest. The duration of this phase was from the beginning of September to the beginning of October, 2023.

### Phase II (program planning)

The training program’s content has been established throughout this phase by reviewing present and past literature, including textbooks, scientific articles in magazines, and internet searches, as well as the outcomes of the pretest evaluation. The researcher began the process of designing and constructing the green management training program to ensure that it was suitable for implementation. The duration of this phase was from the beginning of October to the end of November, 2023.

### Phase III (program implementation)

The training program has been conducted in small groups for the staff nurses. Various teaching methods have been utilized, including lectures, group discussions, brainstorming, role-playing, clinical scenarios, and papers that have been prepared by the researcher & distributed to all staff nurses. The duration of this phase was from the beginning of December 2023 to the end of January 2024.

### Phase IV (post program evaluation)

The influence of the green management training program on staff nurses’ knowledge, green behavior, & organizational citizenship behavior has been assessed through a posttest shortly following the end of the program implementation utilizing the same instruments for data collection as in the preliminary phase. This phase continued from the commencement of February 2024 to the commencement of March 2024. Following the posttest evaluation, a monitoring test has been administered three months later, utilizing the same instruments for data collection as in the preliminary phase, to evaluate the impact of the green management training program on the organizational citizenship behavior of staff nurses. This phase extended from the commencement of March 2024 to the conclusion of June 2024.

### Statistical design

The data was analyzed using the Statistical Package for the Social Sciences (SPSS Inc., version 22; IBM Corp., Armonk, NY, USA) on a personal computer. Descriptive statistics were employed to outline participants’ demographic details. Categorical variables were presented as frequencies, percentages, and Mean SD. A correlation coefficient “Pearson correlation” is a numerical measure of some type of correlation, meaning a statistical relationship between two variables. Cronbach’ alpha to assess internal consistency of the developed tool. Statistical significance was considered at p-value < 0.05. ANOVA, or Analysis of Variance, is a statistical method used to compare the means of three or more measures. Linear regression is a statistical method used to model the relationship between a dependent variable and one or more independent variables.

## Results

### Distribution of the studied staff nurses’ personal and job characteristics (*N* = 85)

Table 1 illustrates that the most of staff nurses (49.4%) were aged between 30 and 40 years, with an average age of 35.7 years with a mean (4.9). Most of them were married (74.1%), and a significant portion hold qualifications from a Nursing Technical Institute (71.8%). Regarding experience, half of staff nurses, 50.6%, had 10–15 years of nursing experience with an average of 12.9 years with a mean (2.6), while 36.5% had less than 3 years of experience in their current department with a mean (3.98 ± 1.13). Only 14.1% had attended training courses on green management.

**Table 1:** Distribution of the studied staff nurses’ personal and job characteristics (number = 85)

Personal - job characteristics	*N*	%
Age:		
< 30 years	20	23.5
30–40 years	42	49.4
> 40 years	23	27.1
**Mean (SD)**	35.7 (4.9)	
**Marital Status**:		
Married	63	74.1
Unmarried	22	25.9
**Qualification**:		
Diploma in Nursing	13	15.3
Nursing Technical Institute	61	71.8
Bachelor of Nursing	11	12.9
**Number of years of experience in nursing**:		
< 10 years	22	25.9
10–15 years	43	50.6
> 15 years	20	23.5
**Mean (SD)**	12.9 (2.6)	
**Number of years of experience in the department**:		
< 3 years	31	36.5
3–5 years	24	28.2
> 5 years	30	35.3
**Mean (SD)**	3.98 (1.13)	
**Attended training courses on green management**:		
Yes	12	14.1
No	73	85.9

SD: Standard deviation

### Staff nurses’ total knowledge level regarding green management throughout program phases (*n* = 85)

Table 2 clarifies that a minority of staff nurses had satisfactory green management knowledge at the pre-program phase. As observed, at the post- program phase, the staff nurse’s satisfactory green management knowledge was improved and marked to be ranged between (84.7% and 97.6%) in all knowledge dimensions. While some improvement (81.2% and 94.1%) occurred in the follow-up phase. Also, there were highly statistically significant differences between all dimensions satisfactory level as well as the total staff nurses’ green management knowledge at post-program phase (*p* < 0.001), while there were statistically significant differences at the follow-up program phase.

**Table 2:** Staff nurses’ total knowledge level regarding green management throughout program phases (*n* = 85)

Staff nurses’ green management satisfactory knowledge (60% +)	Pre		Post		Follow-up		Cochran’s Q Test	
	*N*	%	*N*	%	*N*	%	Test	*p*. value
Green management in hospitals: Why do we need sustainability?	10	11.8	74	87.1	70	82.4	13.557	0.002**
The concept of green behavior and enhancing environmental awareness among nurses:	11	12.9	76	89.4	73	85.9	14.992	0.000**
The most important principles of green management	13	15.3	79	92.9	76	89.4	15.600	0.000**
Sustainability in health care	9	10.6	72	84.7	69	81.2	12.019	0.003**
The most important activities for green behavior and green management	22	25.9	82	96.5	79	92.9	9.887	0.006**
Continuous improvement and development towards green behavior	24	28.2	83	97.6	80	94.1	10.108	0.005**
Organizational citizenship behavior and its importance to the work environment	19	22.4	78	91.8	75	88.2	11.776	0.003**
Effective participation - enhancing environmental organizational citizenship through green management.	21	24.7	77	90.6	74	87.1	13.444	0.000**
Challenges and obstacles to implementing green management in hospitals	13	15.3	75	88.2	72	84.7	9.781	0.006**

* P is significant at < 0.05, ** P is high significant at < 0.01

### Total staff nurses’ green behavior throughout program phases (*n* = 85)

Table 3 reveals that total mean scores of staff nurses’ green behavior dimensions throughout program phases were obviously higher in the post-program phase (90.57 ± 18.6) and follow-up phase (108.98 ± 23.8) than in the pre-program phases (38.81 ± 9.7). Furthermore, there were highly statistically significant differences between total green behavior dimensions throughout program phases (*p* < 0.001).

**Table 3:** Total staff nurses’ green behavior throughout program phases (*n* = 85)

Total staff nurses’ green behavior	Pre		Post		Follow-up		Cochran’s Q Test	
	*N*	%	*N*	%	*N*	%	Test	*p*. value
Influencing Others								
High	4	4.7	49	57.6	61	71.8	12.566	0.002**
Moderate	16	18.8		30.6	19	22.4		
Low	65	76.5	26 10	11.8	5	5.8		
Mean (SD)	7.1 (2.2)		13.99 (4.8)		17.9 (6.1)			
Conserving								
High	5	5.9	51	60	63	74.1		
Moderate	18	21.2		24.7	16	18.8	14.091	0.000**
Low	62	72.9	21 13	15.3	6	7.1		
Mean (SD)	6.9 (2.1)		15.98 (4.7)		18.01 (5.6)			
Avoiding Harm								
High	6	7.1	58	68.2	67	78.8	15.600	0.000**
Moderate	22	25.9		23.5	14	16.5		
Low	57	67.1	20 7	8.3	4	4.7		
Mean (SD)	6.3 (0.98)		14.4 (3.7)		17.8 (5.6)			
Environmental Awareness								
High	5	5.9	60	70.6	68	80	13.811	0.000**
Moderate	25	29.4		24.7	14	16.5		
Low	55	64.7	21 4	4.7	3	3.5		
Mean (SD)	6.37 (1.9)		14.97 (5.7)		18.37 (6.2)			
Taking Initiative								
High	4	4.7	53	62.4	61	71.8		
Moderate	21	24.7		30.5	20	23.5	11.490	0.003**
Low	60	70.6	26 6	7.1	4	4.7		
Mean (SD)	6.4 (2.1)		14.86 (4.3)		18.05 (5.6)			
Working Sustainably								
High	3	3.5	57	67.1	65	76.5	12.006	0.002**
Moderate	32	37.7		23.5	14	16.5		
Low	50	58.8	20 8	9.4	6	7		
Mean (SD)	5.48 (1.3)		15.83 (3.9)		18.36 (5.8)			
Total								
High	5	5.9	55	64.7	61	71.8		
Moderate	19	22.3	22	25.9	18	21.2	16.701	0.000**
Low	61	71.8	8	9.4	6	7		
Mean (SD)	38.81 (9.7)		90.57 (18.6)		108.98 (23.8)			

* P is significant at < 0.05, ** P is high significant at < 0.01

### Total staff nurses’ organizational citizenship behavior level throughout program phases (*n* = 85)

Table 4 reveals that total organizational citizenship behavior dimensions mean scores among staff nurses throughout program phases were obviously increased in the post-program phase (80.12 ± 13.7) and the follows-up phase (93.16 ± 17.8) than preprogram (32.5 ± 4.6). Furthermore, there were highly statistically significant differences between total organizational citizenship behavior dimensions throughout program phases (*p* < 0.001).

**Table 4:** Total staff nurses’ organizational citizenship behavior level throughout program phases (*n* = 85)

Total staff nurses’ organizational citizenship behavior	Pre		Post		Follow-up		Friedman test	
	*N*	%	*N*	%	*N*	%	Test	p. value
Altruism (Selflessness) 5								
High	6	7.1	59	69.4	67	78.8	9.812	0.003**
Moderate	24	28.2	18	21.2	14	16.5		
Low	55	64.7	8	9.4	4	4.7		
Mean (SD)	5.65 (0.99)		20.08 (3.6)		23.36 (4.1)			
Civility (courtesy) 4								
High	5	5.9	61	71.8	70	82.4		
Moderate	26	30.6	15	17.6	10	11.8	15.440	0.000**
Low	54	63.5	9	10.6	5	5.8		
Mean (SD)	7.42 (1.6)		15.94 (4.3)		18.9 (3.7)			
Sportsmanship 3								
High	4	4.7	63	74.1	68	80	16.071	0.000**
Moderate	30	35.3	12	14.1	10	11.8		
Low	51	60	10	11.8	7	8.2		
Mean (SD)	5.65 (1.8)		12.37 (3.4)		13.97 (2.99)			
Civic virtue (civilized behavior) 5								
High	7	8.2	60	70.6	63	74.1		
Moderate	25	29.4	10	11.8	15	17.6	15.441	0.000**
Low	53	62.4	15	17.6	7	8.3		
Mean (SD)	8.69 (2.7)		19.83 (4.7)		23.07 (6.9)			
Conscientiousness (Awareness of conscience) 3								
High	5	5.9	58	68.2	67	78.8	12.990	0.002**
Moderate	20	23.5	18	21.2	12	14.1		
Low	60	70.6	9	10.6	6	7.1		
Mean (SD)	5.09 (1.8)		11.99 (3.4)		13.93 (4.5)			
Total Organizational citizenship behavior								
High	6	7.1	60	70.6	64	75.3		
Moderate	30	35.3	17	20	16	18.8	15.301	0.000**
Low	49	57.6	8	9.4	5	5.9		
Mean (SD)	32.5 (4.6)		80.12 (13.7)		93.16 (17.8)			

* P is significant at < 0.05, ** P is high significant at < 0.01

### Correlation between staff nurses’ knowledge about green management, their green behavior level and organizational citizenship behavior(*n* = 85)

Table 5 shows that there was a positive, highly statistically significant correlation (*r* = 0.399, *p* < 0.001) between green management knowledge, green behavior level and organizational citizenship behavior among staff nurses.

**Table 5:** Correlation between staff nurses’ knowledge about green management, their green behavior level and organizational citizenship behavior(*n* = 85)

Parameter		Employee green behavior	Organizational citizenship behavior	Nurses’ knowledge
Employee green behavior	r. p.			
Organizational citizenship behavior	r. p.	0.499 0.000**		
Nurses’ knowledge	r. p.	0.416 0.001**	0.399 0.002**	

** P is high significant at < 0.001 r: Pearson coefficient

### Multiple linear regression model for organizational citizenship behavior at post program phase (*n* = 85)

Table 6 shows, organizational citizenship behavior level was positive dependent for both the green behavior level and total knowledge at the post program phase. The model explains 63% of the variance in organizational citizenship behavior (R² = 0.63), and the overall regression is significant (F = 13.444, *p* < 0.05).

**Table 6:** Multiple linear regression model for organizational citizenship behavior at post program phase (*n* = 85)

Model		Unstandardized Coefficients	standardized Coefficients		
		B	Β	T	*P*. value
Employee green behavior		0.678	0.518	7.801	< 0.01**
Total knowledge		0.452	0.390	6.331	< 0.01**
Model	R^2^	Df.	F	P. value	
Regression	0.63	1	13.444	0.000**	

a. Dependent Variable: Organizational citizenship behavior

b. Predictors: (constant): Employee green behavior, Nurses Knowledge

** P is high significant < 0.01 F, p: f and p values for the model

B: Unstandardized Coefficients R2: Coefficient of determination

t: t-test of significance Beta: Standardized Coefficients

SE: standard error

## Discussion

Green management in hospitals refers to the implementation of environmentally sustainable policies and procedures in the operation and management of healthcare facilities [30]. OCB encompasses voluntary actions by workers that extend beyond their formal duties, fostering a positive work environment and advancing organizational effectiveness [31]. As sustainability gains prominence, green management practices are becoming essential to align environmental responsibility with healthcare goals. This study aims to evaluate the impact of GMTP on the OCB of staff nurses. Given the pivotal role that nurses play, improving their awareness and practices related to green management can significantly influence organizational efficiency and environmental sustainability. Through targeted training, the study explores whether incorporating green principles into nursing practices can encourage behaviors that support long-term organizational objectives, resulting in enhanced patient care and environmental stewardship [32].

Concerning personal and job characteristics, the study revealed a majority of staff nurses aged between 30 and 40 years, with an average of 35.7 years. Most of them were married, and a significant portion 71.8% hold qualifications from a nursing technical institute. More than half of staff nurses had 10–15 years of nursing experience, with an average of 12.9 years, while more than one third had below 3 years of experience in their present department. This outcome is maintained by, Elgarfet al. [33] found that more than 50% of the examined nursing staff were between thirty to forty years old. Similarly, Abd-Elhamid and Gaber [34] noted that there was a significant relation between age, attending training, qualification, and green behavior level. Also, the distribution of personal data of the studied nursing managers: more than two-thirds of the studied participants were female, their age mean was about 34 years old, and regarding their qualifications, more than half of them graduated from a technical institute, and finally, all of them did not receive any training program regarding green management.

Regarding the total staff nurses’ knowledge regarding green management throughout program phases, the present study revealed that fewer than one-tenth of the staff nurses had a satisfactory knowledge level of sustainability in health care prior to the program’s implementation. There was a significant improvement in staff nurses’ knowledge immediately after the program. However, a slight decline was observed during the follow-up phase, although the knowledge levels remained significantly higher compared to the pre-program phase. This could be due to the fact that the majority of staff nurses had never taken a green management training.

This finding was in line with, Saleh et al. [2] reported a significant improvement in the level of green management among nursing managers. Also, indicated that a large proportion of nursing managers who had a low level of green management prior to the intervention were able to transition to a high level of green management. Furthermore, the sustained high level of green management among nursing managers at the follow-up stage indicates the effectiveness of the intervention. Also, supported by Garg & Dewan [35] revealed that before implementation of green management program, the majority of the nursing staff had an unsatisfactory knowledge level about green management, after the program’s implementation, almost all of them had a satisfactory level of knowledge.

According to the total staff nurses’ green behavior level, the result of the present study revealed that high enhancement in staff nurses’ green behavior mean scores at the post-program and follow-up phases in comparison with the pre-program phase. From the researcher’s point of view, this may be due to program intervention that included sustainable practices, enhancing the skills, knowledge, and perception of green behavior of staff nurses, and equipping them with the tools and resources needed to effectively lead and contribute to the improvement of organizational sustainability. Also, this finding is in agreement with, Aboramadan [36] noted that most of the studied sample had good green behavior and was positively contribute to employee green outcomes. Also, this finding is in the same line with, Muafi [37] reported there was a positive and significant effect between environmental awareness and green behavior mediated by employee well-being, and the higher the environmental knowledge, the employees’ welfare will increase, which causes better green behavior.

With regards to total staff nurses’ organizational citizenship behavior, the present investigation results illustrated that there was a high enhancement in staff nurses’ organizational citizenship behavior mean scores at the post-program and follow-up phases in comparison with the pre-program phase. The outcome of current investigation is in line with, Mohamed Ali et al. [38] found that proper commitment to an organization increases OCB on employees, as good organizational commitment is due to satisfaction and trust in the organization. Also, employees with high commitment are highly responsible and loyal toward their job, all these factors will increase OCB among nursing staff. Also, Mostafa& Hasaballah [39] indicated highly improved organizational citizenship behavior among nursing personnel. Additionally, Azizah et al. [40] found that most of the application of altruism is high for hospital services, conscientiousness, and sportsmanship and courtesy. Also, recent study suggested that strategies to enhance job satisfaction, such as reducing work-related stress and fostering supportive work environments [41].

The present investigation results illustrated that there is a significant correlation between staff nurses’ knowledge and staff nurses’ green behavior, as well as between nurses’ knowledge and organizational citizenship behavior. From the researcher’s perspective, this improvement could be attributed to the implementation of the training program and the acquisition of various knowledge and initiatives by staff nurses throughout the program concerning green management and their practical application in the workplace. The outcomes were in the same line with, Aboramadan [36] showed a significant association among worker green behavior and organizational citizenship behavior. Also, it is implied that employees with higher levels of green work engagement are more prone to having a trustful and quality exchanges with their organization, which would ultimately encourage employees to display positive outcomes such as green outcomes and enhancing organizational citizenship.

According to the best-fitting multiple linear regression for OCB level at the monitoring program, the current investigation illustrated that OCB was positively dependent on both green behavior level and total knowledge at the follow-up phase. From the researcher’s perspective, this might be related to the efficiency of the training program for improving staff nurses’ knowledge and enhancing green behavior practices, adapting with sustainability throughout the training program, which led to the development of the spirit of OCB between staff nurses at the workplace and decreasing environmental impact. The outcomes were in agreements with, Galván-Mendoza et al. [42] showed that environmental knowledge has a positive and statistically significant influence on perceived behavioral control and worker green behavior. Additionally, it has been detected that the perceived behavioral control parameter had a positive and statistically significant influence on the worker green behavior of women workers. Also, Fawehinmi et al. [43] showed that green human resources management has a positive impact on employee green behavior, nurse empowerment, and green job creation, as well as organizational environmental citizenship. This finding gives a theoretical implication in terms of ability, motivation, and opportunity theory.

### Implications of the study

The findings of the study suggest that implementing targeted training programs can significantly improve staff nurses’ knowledge of green management, which can directly inform hospital policies on sustainability. Hospitals can adopt policies that promote environmentally conscious actions in daily nursing routines, encouraging sustainable behaviors across the organization. Policies should also focus on identifying and applying effective interventions to raise environmental awareness among staff, enhancing their understanding of sustainability practices. Additionally, hospitals can prioritize sustainable healthcare practices aimed at reducing the environmental impact of their facilities. To foster a culture of sustainability, hospital policies can integrate eco-friendly values into organizational practices and promote organizational citizenship behavior, motivating staff to actively participate in sustainability initiatives.

## Conclusion

In light of the outcomes of the present investigation, it can be concluded that staff nurses in the study setting initially exhibited unsatisfactory knowledge regarding green management, insufficient practice of employee green behavior, and low levels of organizational citizenship behavior during the pre-program phase. However, highly statistically significant improvements were detected in staff nurses’ knowledge of green management, employee green behavior, and organizational citizenship behavior after the implementation of the green management training program. A highly statistically significant, strong positive correlation was found between the total green management knowledge and total organizational citizenship behavior. Finally, “The study findings support the hypothesis that a green management training program significantly enhances staff nurses’ knowledge, green behavior, and OCB.”

Based on these findings, it is recommended that future research explore the impact of providing workshops on organizational sustainability and guidelines for nurses’ green behavior. Additionally, strategies should be developed for nurses and their leadership to implement strengths-based feedback methodologies that promote sustainable practices and organizational citizenship behavior.”

### Limitations of the study

The study was conducted in a single healthcare setting, which may limit the generalizability of the findings to other institutions or settings. Additionally, the study did not assess the long-term sustainability of the improvements observed, which is important for understanding whether the positive changes will persist over time. Factors influencing the successful implementation of green management practices in healthcare settings, such as organizational support or resource availability, were not explored.

## Data Availability

No datasets were generated or analysed during the current study.

## Abbreviations

GMTP: Green Management Training Program
OCB: Organizational Citizenship Behavior
VBN: Value-Belief-Norm
NEP: New Ecological Paradigm
